# Biogenic Gold Nanoparticles as Potent Antibacterial and Antibiofilm Nano-Antibiotics against *Pseudomonas aeruginosa*

**DOI:** 10.3390/antibiotics9030100

**Published:** 2020-02-27

**Authors:** Syed Ghazanfar Ali, Mohammad Azam Ansari, Mohammad A. Alzohairy, Mohammad N. Alomary, Sami AlYahya, Mohammad Jalal, Haris M. Khan, Sarah Mousa Maadi Asiri, Wasim Ahmad, Abbas Ali Mahdi, Ahmed M. El-Sherbeeny, Mohammed A. El-Meligy

**Affiliations:** 1 Department of Microbiology, Nanotechnology and Antimicrobial Drug Resistance Research Laboratory, Jawaharlal Nehru Medical College and Hospital, Aligarh Muslim University, Aligarh 202001, India; jalalmicro1981@gmail.com (M.J.);; 2 Department of Epidemic Disease Research, Institute for Research and Medical Consultations (IRMC), Imam Abdulrahman Bin Faisal University, P.O. Box 1982, Dammam 31441, Saudi Arabia; 3 Department of Medical Laboratories, College of Applied Medical Sciences, Qassim University, Qassim 51431, Saudi Arabia; 4 National Center for Biotechnology, Life Science and Environmental Research Institute, King Abdulaziz City for Science and Technology, P.O. Box 6086, Riyadh 11442, Saudi Arabia; malomary@kacst.edu.sa; 5 National Center for Biotechnology, King Abdulaziz City for Science and Technology, P.O. Box 6086, Riyadh 11442, Saudi Arabia; salyahya@kacst.edu.sa; 6 Department of Biophysics, Institutes for Research and Medical Consultations (IRMC), Imam Abdulrahman Bin Faisal University, Dammam 31441, Saudi Arabia; smasiri@iau.edu.sa; 7 Department of Pharmacy, Mohammad Al-Mana College for Medical Sciences, Dammam 34222, Saudi Arabia; wasimahmadansari@yahoo.com; 8 Departments of Biochemistry, King George Medical University, Lucknow 226003, India; abbasalimahdi@gmail.com; 9 Industrial Engineering Department, College of Engineering, King Saud University, P.O. Box 800, Riyadh 11421, Saudi Arabia; aelsherbeeny@ksu.edu.sa; 10 Advance Manufacturing Institute, King Saud University, Riyadh 11421, Saudi Arabia; melmeligy@ksu.edu.sa

**Keywords:** biofilm, gold nanoparticles, Con A-FITC, nano-antibiotics, CLSM, *Pseudomonas aeruginosa*, *Tinospora cordifolia*

## Abstract

Plant-based synthesis of eco-friendly nanoparticles has widespread applications in many fields, including medicine. Biofilm—a shield for pathogenic microorganisms—once formed, is difficult to destroy with antibiotics, making the pathogen resistant. Here, we synthesized gold nanoparticles (AuNPs) using the stem of an Ayurvedic medicinal plant, *Tinospora cordifolia*, and studied the action of AuNPs against *Pseudomonas aeruginosa* PAO1 biofilm. The synthesized AuNPs were characterized by techniques such as ultraviolet-visible spectroscopy, Fourier-transform infrared (FTIR) spectroscopy, energy-dispersive X-ray diffraction, X-ray diffraction, scanning electron microscopy (SEM), and transmission electron microscopy. The AuNPs were spherically shaped with an average size of 16.1 nm. Further, the subminimum inhibitory concentrations (MICs) of AuNPs (50, 100, and 150 µg/mL) greatly affected the biofilm-forming ability of *P. aeruginosa*, as observed by crystal violet assay and SEM, which showed a decrease in the number of biofilm-forming cells with increasing AuNP concentration. This was further justified by confocal laser scanning microscopy (CLSM), which showed irregularities in the structure of the biofilm at the sub-MIC of AuNPs. Further, the interaction of AuNPs with PAO1 at the highest sub-MIC (150 µg/mL) showed the internalization of the nanoparticles, probably affecting the tendency of PAO1 to colonize on the surface of the nanoparticles. This study suggests that green-synthesized AuNPs can be used as effective nano-antibiotics against biofilm-related infections caused by *P. aeruginosa.*

## 1. Introduction

*Pseudomonas aeruginosa* is an opportunistic pathogen and has become a model organism for biofilm research. According to the US National Healthcare Safety report (2007), *P. aeruginosa* was found to be the second most frequent cause of ventilator-related pneumonia, the sixth most recurrent pathogen and the seventh most common cause of catheter-associated bloodstream infection in the ~28,000 cases of nosocomial infections that were recorded in 463 hospitals over a period of 22 months [1,2]. The ability of *P. aeruginosa* to form biofilms is a major virulence factor and is associated with nosocomial infections. The chief characteristic of this organism is its ability to develop resistance against antibiotics [3]. Modification of enzymes, target-site mutation, and efflux pumps are important factors that contribute to the development of antibiotic resistance [4].

This bacterium can form biofilms on different surfaces, such as mucus plugs of cystic fibrosis patients, contaminated catheters, and contact lenses [5]. Biofilms, formed by some microorganisms, are complex communities covered by self-made exopolysaccharide (EPS) layers, which are irreversibly attached to these surfaces [6]. A mature biofilm has a suitable bacterial communication system inside the EPS layers. When a bacterial population reaches a threshold concentration, the bacteria sense it and start activating or repressing certain target genes by quorum sensing (QS). The mechanism of QS is directly involved with the formation of biofilms in different bacterial species, including *P. aeruginosa* [7,8].

A biofilm develops through five stages: (i) firm attachment of the bacterial cells to the surface, (ii) irreversible attachment of the cells to the surface, (iii) development of new colonies, (iv) secretion of EPS, and (v) dispersal [9]. The biofilm plays a major role in developing resistance towards antimicrobial agents [10]. Once these microorganisms develop resistance, they become more infectious and difficult to eradicate.

Nanotechnology, an emerging field of science, has many applications in different fields including agriculture, electronics, biomedicine, etc. The application of nanoparticles and their conjugates in antimicrobial therapy is being widely studied [11]. Gold nanoparticles (AuNPs) have been used as antibacterial, anti-HIV, antitumor, antimalarial, and antibiofilm substances, [12] besides being used in biosensors, DNA labeling, and vapor sensing [13,14,15]. Physical, chemical, and biological methods of the synthesis of nanoparticles have been developed. The biological method, or the green method, of synthesis is advantageous over the chemical and physical methods [16,17,18] because it eliminates the chances of formation of toxic byproducts and utilizes plant parts, including leaves, bark, etc., for synthesis [19,20,21]. Different reports of the synthesis of AuNPs, using lemongrass, tealeaves, and human cells, have been documented in the literature [22,23,24].

*Tinospora cordifolia* (Willd.) Miers is an Ayurvedic medicinal plant, which has been used since ancient times for the treatment of different diseases. The stem of *T. cordifolia* possesses antidiabetic effects because it regulates the blood glucose level in the body [25]. The extract obtained from the roots of this plant has the ability to scavenge free radicals generated during aflatoxicosis [26]. The aqueous extracts of the stem and leaves of *T. cordifolia* have been shown to be effective against lead toxicity because they improve hematological values [27]. The stem extract alone can decrease blood urea concentration in uremia, relieve urinary infections, and dissolve urinary calculi [28,29]. Methanolic extract of the stem, besides being antimicrobial [30], also possesses antioxidant activity, increasing the activities of erythrocyte membrane lipid peroxidation and catalase when administered orally. It also lowered the activities of superoxide dismutase (SOD) and glutathione peroxidase (GPx) in alloxan-induced diabetic rats [31,32,33]. Some studies have shown that the use of *T. cordifolia* extract gives better results than that of doxorubicin [34].

To ensure that our process remained eco-friendly, we selected a medicinally important Ayurvedic plant, *T. cordifolia*, for the synthesis of AuNPs and showed the effects of AuNPs against the biofilm formed by *P. aeruginosa.*

Since biofilm-mediated infections cannot be overcome by antibiotics, we thought of a newer approach to eradicate the biofilm. Green nanotechnology, which is based on the formation of nanoparticles using medicinal plants, could be the answer because it exploits the medicinal properties of plants. Thus, our study is based on green synthesis of AuNPs using *T. cordifolia*, an Ayurvedic medicinal plant, and the characterization of AuNPs using different techniques such as ultraviolet-visible (UV-Vis) spectroscopy, Fourier-transform infrared (FTIR) spectroscopy, energy-dispersive X-ray (EDX) diffraction, X-ray diffraction (XRD), scanning electron microscopy (SEM), and transmission electron microscopy (TEM).

## 2. Results

### 2.1. Characterization of Nanoparticles

**Figure 1:**
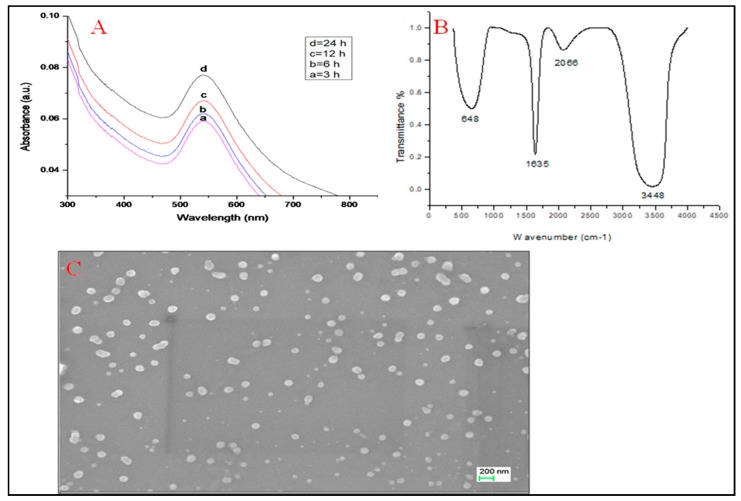
Characterization of synthesized gold nanoparticles. (**A**) Ultraviolet-visible (UV-Vis) and (**B**) Fourier-transform infrared (FTIR) spectra of green-synthesized gold nanoparticles (AuNPs). (**C**) Scanning electron microscopy (SEM) analysis showing different nanoparticles scattered without agglomeration.

Stepwise representation of green synthesis of AuNPs using the aqueous stem extract of *T. cordifolia* is shown in supplementary Figure S1. UV-Vis spectra of *T. cordifolia* AuNPs (TC-AuNPs) showed strong surface plasmon resonance (SPR) at 542 nm, along with a pointed band (Figure 1A). The pointed band is suggestive evidence for the formation of spherical and small-sized nanoparticles.

**Figure 2:**
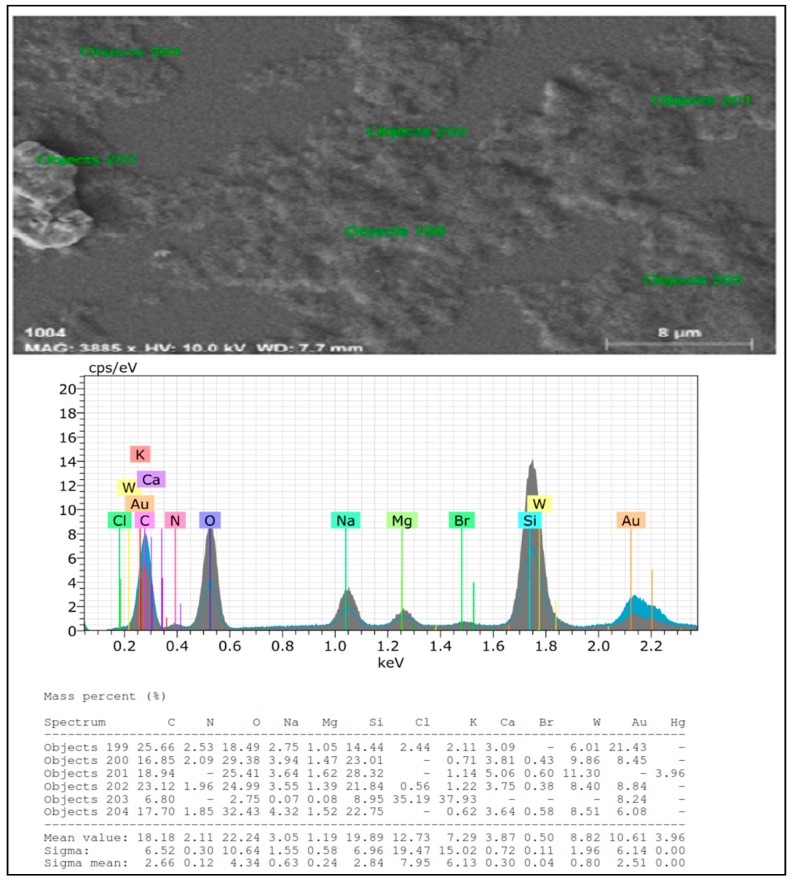
Energy-dispersive X-ray (EDX) spectra of green-synthesized AuNPs.

FTIR spectra of TC-AuNPs displayed four different peaks at wave numbers 648, 1635, 2066, and 3448 cm^−1^, which probably correspond to C-C, C=O-, N-H, and OH stretching, respectively (Figure 1B). These bonds are involved in the formation of nanoparticles. Figure 1C is a representative image of SEM, showing uniform distribution of nanoparticles without aggregation and clumping, whereas Figure 2 shows an elemental composition, shown by EDX diffraction, analyzed at six different locations, in a particular slide mentioned as objects. It showed a range of 21.43% to 6.08% of gold at different sites (Figure 2).

**Figure 3:**
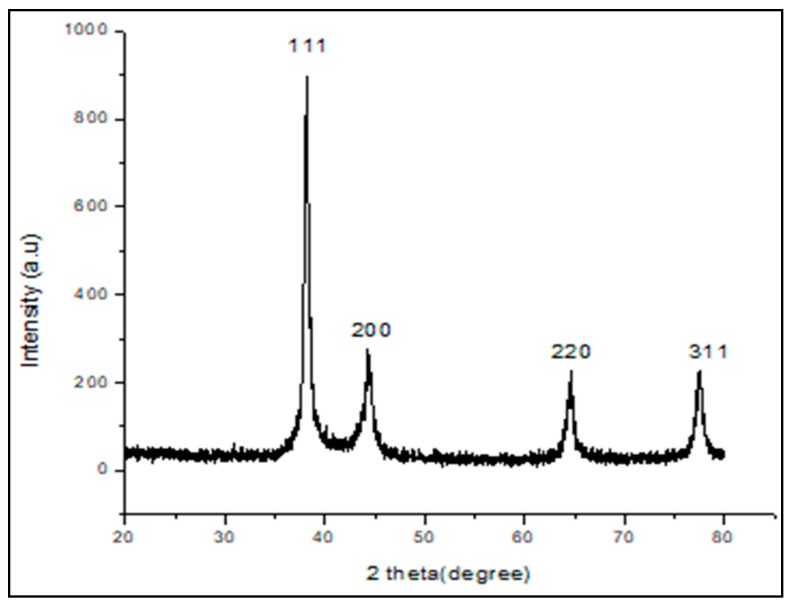
X-ray diffraction (XRD) analysis of green-synthesized AuNPs.

XRD analysis represented the crystalline nature of nanoparticles. The respective diffraction peaks at 38.1°, 44.3°, 64.71°, and 77.3°, relating to (111), (200), (220), and (311) facets of the face-centered cubic (FCC) crystal lattice, corresponded to pure gold (Figure 3) (JCPDS card no 04-0784). The average size of the crystal *d* of TC-AuNPs was calculated by the Scherrer equation:

d = Kλ/βcosθ,(1)

where K is the shape factor between 0.9 and 1.1 (CuKα = 1.542Å), β is the full width half-maximum of the prominent line (111) in radians, and θ is the position (38.1°) of that line in the pattern. The average size of the crystal particle was found to be 17.6 nm (Figure 3).

**Figure 4:**
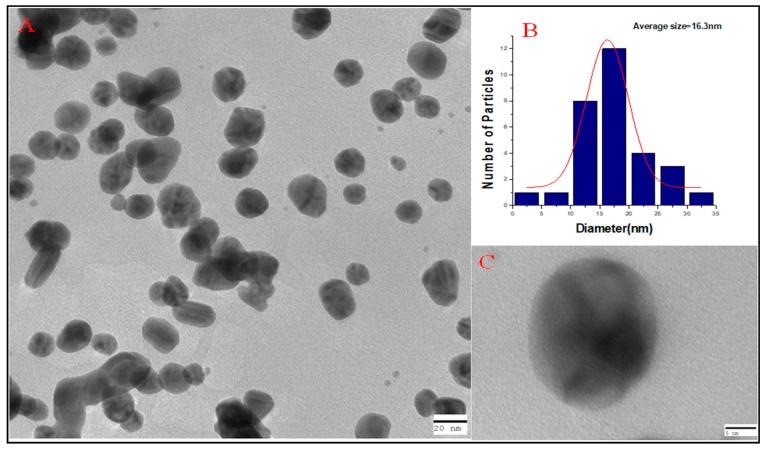
Electron microscopic analysis of green-synthesized gold nanoparticles (AuNPs). (**A**) Transmission electron microscopy (TEM) image representing different sizes of green-synthesized AuNPs. (**B**) Histogram showing the average particle size as calculated by ImageJ. (**C**) Individual AuNP at greater magnification, indicating the spherical nature of the particle.

TEM analysis depicted the morphology and size of nanoparticles. Figure 4A shows that most of the nanoparticles that were polydispersed were roughly spherical in nature. The average particle size of TC-AuNPs, as analyzed by the histogram, was 16.3 nm (Figure 4B). Figure 4C shows an individual nanoparticle at greater magnification, indicating the spherical nature of AuNPs.

The minimum inhibitory concentration (MIC) of TC-AuNPs against PAO1 was found to be 1000 µg/mL. The sub-MIC, which did not inhibit growth, was 150 µg/mL; therefore, three different sub-MIC values (50, 100, and 150 µg/mL) were considered for SEM and confocal laser scanning microscopy (CLSM) analysis of antibiofilm activity of TC-AuNPs. 

### 2.2. Antibiofilm Potential as Characterized by SEM, Crystal Violet Assay, and CLSM

**Figure 5:**
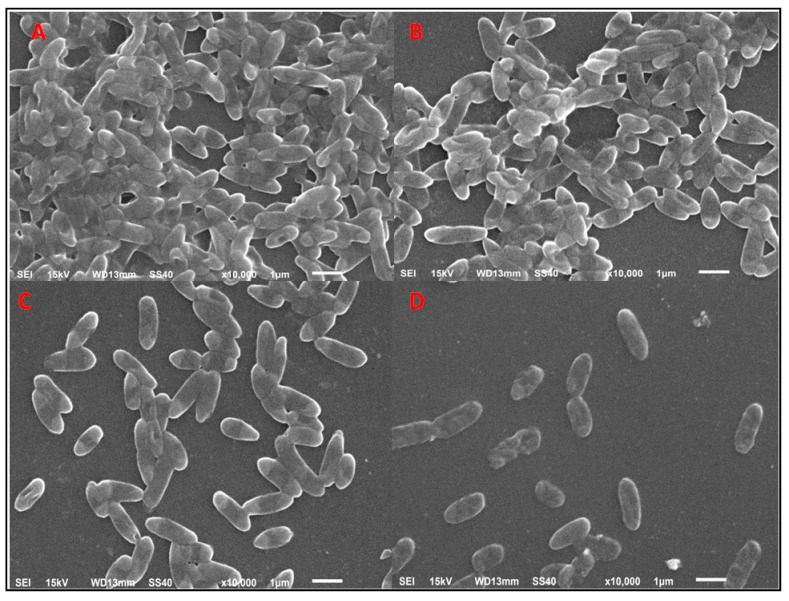
Scanning electron microscopic analysis of biofilm structure. SEM images of the biofilms formed on the glass coverslips after 24 h of incubation. (**A**) Control; (**B**), (**C**), and (**D**) treated with 50, 100, and 150 µg/mL of *Tinospora cordifolia* (TC)-AuNPs, respectively.

**Figure 6:**
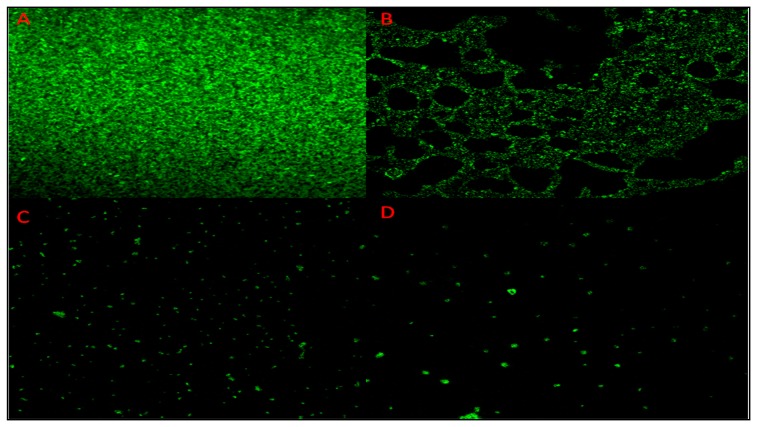
Confocal laser scanning microscopy (CLSM) images of the biofilm formed by *P. aeruginosa* PAO1 on the glass coverslips after 24 h of incubation. (**A**) Control; (**B**), (**C**), and (**D**) treated with 50, 100, and 150 µg/mL of TC-AuNPs, respectively.

SEM, crystal violet assay, and CLSM showed the antibiofilm nature of nanoparticles in a dose-dependent manner. Figure 5 represents the SEM image of bacterial cells after treatment with AuNPs, showing the decrease in the number of cells. The control sample showed that a greater number of cells adhered to the surface (Figure 5A), whereas the subsequent images showed the decrease in the number of biofilm-forming cells with the increase in the concentration of nanoparticles (Figure 5B–D). Bacterial cells with the glycocalyx matrix, which is a prerequisite for the formation of the bacterial biofilm, could not be viewed by SEM. Hence, CLSM analysis was performed. Fluorescent dye concanavalin-A-conjugated fluorescein isothiocyanate (Con A-FITC) bound to the mannose residues, resulting in the green staining of the bacterial glycocalyx. In the CLSM images, it was seen that TC-AuNPs disrupted the biofilm architecture, and no distinct pattern of arrangement of cells could be seen. Further, the dose-dependent reduction of colonies could be clearly observed (Figure 6A–D). The quantitative crystal violet assay was performed to investigate the inhibition of biofilm formation after treatment with different concentrations of AuNPs. It was found that biofilm formation was inhibited up to 59.9% at 150 µg/mL, 36.6% at 100 µg/mL, and 27.1% at 50 µg/mL of AuNPs (Supplementary Table S1).

### 2.3. Localization of TC-AuNPs Inside the Bacterial Cells: TEM Analysis

**Figure 7:**
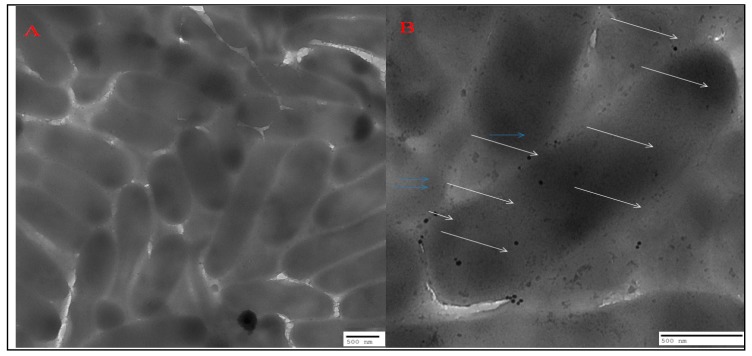
**Ultrastructural changes caused by gold nanoparticles as analyzed by TEM.** (**A**) Images of the control setup of *P. aeruginosa* PAO1 without treatment with nanoparticles. (**B**) Images of the experimental setup of PAO1 treated with 150 µg/mL of AuNPs. Blue arrows indicate nanoparticles outside the cell, whereas white arrows indicate internalized nanoparticles.

Figure 7A represents the TEM images showing the untreated PAO1 cells, and Figure 7B represents cells treated with nanoparticles of the highest MIC (150 µg/mL). These internalized nanoparticles adversely affected the adherence property of the cells, due to which cells could not colonize and were unable to form biofilms.

## 3. Discussion

The change in color from light yellow to dark pink was the first indication of the reduction of AuCl_3_ to AuNPs. This was due to the surface plasmon vibrations with aqueous AuNPs [35]. Rajkumari et al. [36] obtained similar results by obtaining the UV spectra of green-synthesized AuNPs at 550 nm.

FTIR analysis showed the formation of different bonds between the AuNPs and plant extracts. These bonds were the biomolecules involved in capping and responsible for stabilization of individual AuNPs.

SEM and EDX diffraction analysis depicted the distribution of nanoparticles and showed that most of the nanoparticles were spherical in shape and uniformly distributed without aggregation. It further supported that the concentration of *T. cordifolia* extract was enough to reduce gold chloride (AuCl_3_) to its nanoform. It can also be concluded that plant extracts provided enough biomolecules to act as a supporting matrix for the capping and stabilization of gold. Moreover, the nature of nanoparticles so formed was amorphous, as analyzed by XRD. For determining the size of AuNPs, TEM analysis was performed at 80,000× at 200 kV. It represented the different nanoparticles that were polydispersed, and the average size of the AuNPs was found to be 16.3 nm. On further enlargement at 500,000× at 200 kV, an individual particle with a spherical shape was seen, demonstrating that the majority of the particles were spherical. It also supported that the formation of spherical nanoparticles depended on the outstanding capping and stabilizing properties of the extracts [37]. TC-AuNPs decreased the biofilm-forming ability of *P. aeruginosa* in a dose-dependent manner. It was evident from the SEM analysis that the number of biofilm-forming cells decreased with the increase in the concentration of nanoparticles, showing that the cells neither adhered to the surface nor colonized. However, there were certain limitations to the SEM analysis, such as the limitation in detection of EPS and the reduction in the total volume and architecture of the cell during the dehydration process of SEM [38]. Therefore, to view the biofilm and EPS, CLSM analysis was performed using Con A-FITC. CLSM analysis detected the biofilm at different depths nondestructively and maintained its three-dimensional structure [39,40]. Our results also suggested that the cells were unable to colonize due to their inability to secrete EPS. Previous findings by Singh et al. [41] reported the inhibition of QS signaling molecules in PAO1 when silver nanoparticles (AgNPs) at sub-MIC were used. Internalization of mycofabricated AgNPs and their effects on QS-operated virulence factors were reported. Ali et al. [20] also reported similar findings where AgNPs inhibited QS-mediated virulence factors. Our SEM analysis results agreed with previous findings of Samantha et al. [42]. They showed that AuNPs, synthesized using fungus *Laccaria fraterna*, also inhibited biofilm formation in the same manner. Similar results of biofilm inhibition using CLSM were shown by Subhaswaraj et al. [43]. Our findings were also phenotypically similar to those of Rajkumari et al. [36]. They showed the decrease in the biofilm-forming ability of PAO1 using Baicalin-conjugated nanoparticles. Our findings of biofilm inhibition using crystal violet assay agreed with those of Khan et al. [44]. They showed that fucoidan-stabilized AuNPs inhibited biofilm formation. However, the mode of action of AuNPs needs to be studied further, not only phenotypically, but also genetically.

## 4. Materials and Methods

### 4.1. Preparation of the Aqueous Stem Extract of T. cordifolia

The stem of *T. cordifolia* was collected from the nearby area of Aligarh, Uttar Pradesh, India. The outer husk of the upper portion of the stem was removed, and the stem was sun-dried. The dried stem was crushed and ground into a fine powder, and the powder (10 g) was then dissolved in sterile water (100 mL). After 24 h, the aqueous extract was filtered and purified by centrifugation; finally, the obtained aqueous stem extract was used for the synthesis of AuNPs.

### 4.2. Synthesis of TC-AuNPs

The aqueous stem extract (10 mL) was mixed with the aqueous solution of 1 mM AuCl_3_ (90 mL). The color of the solution changed from yellow to pink within 1 h and then to dark pink within 24 h. This indicated the reduction process and formation of TC-AuNPs.

### 4.3. Characterization of TC-AuNPs

#### 4.3.1. UV-Vis Spectroscopy

The formation of TC-AuNPs was characterized by the UV-Vis spectrophotometer (PerkinElmer Lambda 25, Shelton, CT, USA) in the range of 250–800 nm. The samples were briefly placed in the cuvette, and UV-Vis spectrum was measured at different time intervals.

#### 4.3.2. FTIR Spectroscopy

Complex bonds formed due to the interaction between biomolecules present in the extract and gold particles were analyzed by FTIR (PerkinElmer, CT, USA) spectroscopy in the range of 4000–400 cm^−1^ at room temperature. The adsorption spectrum displayed different peaks, which corresponded to the various bonds formed [20].

#### 4.3.3. SEM and EDX Diffraction

SEM (Jeol JSM-6510LV, Tokyo, Japan) was used to determine the distribution of nanoparticles, whereas EDX (Bruker) diffraction was used to analyze the elemental composition. Thin films of nanoparticles were formed on glass coverslips by spreading the nanoparticles, and the samples were then coated with gold. The films were analyzed at an accelerating voltage of 15 kV using a scanning electron microscope equipped with an EDX. 

#### 4.3.4. XRD Pattern of TC-AuNPs 

The crystalline nature of TC-AuNPs was confirmed by XRD (Bruker D8 Diffractometer, GmbH, Karlsruhe, Germany) using Cu_Ka_ radiation (λ = 1.54056 Å) in the range of 20° ≤ 2θ ≤ 80° at 40 keV. The samples were briefly placed for X-ray, and the peaks obtained were further analyzed.

#### 4.3.5. TEM

TEM (Jeol 2100, Tokyo, Japan) was used to determine the size and shape of the green-synthesized TC-AuNPs. The synthesized TC-AuNPs were briefly placed on the copper grid (Sigma-Aldrich, St. Louis, MO, USA) and allowed to dry. After drying, the samples were placed in the transmission electron microscope, which illuminated the sample with electronic radiation under vacuum. The electron beam, which transmitted through the sample, allowed for the detection of the sample [45].

### 4.4. Bacterial Strain

*P. aeruginosa* PAO1 was used as the model organism for assessment of antibacterial and QS-mediated antibiofilm activity.

### 4.5. MIC of TC-AuNPs

MIC was determined by using a macrobroth dilution method, following the procedure described by Ansari et al. [46]. Initially, PAO1 was allowed to grow on nutrient agar plates, and the plates were incubated at 37 °C for 24 h. Fresh colonies from nutrient agar plates were used to inoculate the nutrient broth, which was further incubated at 37 °C for 24 h. A fresh culture of *P. aeruginosa* (2 × 10^6^ CFU/mL), grown overnight, was used to inoculate each tube. Each tube was twofold serially diluted with TC-AuNPs at different concentrations then incubated again at 37 °C for 24 h.

### 4.6. Characterization of Antibiofilm Potential of TC-AuNPs Using SEM

Antibiofilm potential of TC-AuNPs was assessed using SEM. The biofilm was briefly allowed to develop on glass coverslips, seeded in 4 mL Brain heart infusion (BHI) broth (5% sucrose) in a 12-well microtiter plate. Mid exponential growth phase culture of PAO1 (100 µL) was then inoculated along with different concentrations of AuNPs [46]. A well without nanoparticles was treated as the control. The plate was incubated at 37 °C for 24 h. The glass coverslips were removed from the wells after incubation, washed with phosphate buffer saline (PBS), and fixed with 2.5% glutaraldehyde. After fixation, the samples were washed by a series of alcohol samples of different concentrations (30, 50, 70, 90, and 100%), each for 5 min at room temperature. Finally, after one more washing with PBS, the cells were mounted on aluminum stubs, and gold coating was performed. The effects of AuNPs on the biofilm were visualized using a scanning electron microscope (Jeol JSM-6510 LV, Tokyo, Japan) with an accelerating voltage of 15 kV.

### 4.7. Characterization of Antibiofilm Activity of TC-AuNPs Using CLSM

Antibiofilm potential of green-synthesized TC-AuNPs against *P. aeruginosa* was characterized by CLSM as described previously [47,48]. The media (BHI + 5% sucrose) (4 mL), along with glass coverslips, were amended in each well of a 12-well microtiter plate. Further, varying concentrations of AuNPs, along with the mid exponential growth phase culture of *P. aeruginosa*, grown overnight, were inoculated in each well. The control well was not amended with nanoparticles. The plates were incubated at 37 °C for 24 h. After incubation, the glass coverslips were removed, washed with PBS, and then stained with Con A-FITC (Sigma-Aldrich, St. Louis, MO, USA), according to the protocol described by Ansari et al. [47]. The effects of nanoparticles on biofilm structures were examined by a Fluoview FV1000 Espectral Olympus confocal scanning laser microscope (Olympus Latin America, Miami, FL, USA).

### 4.8. Characterization of Antibiofilm Activity of TC-AuNPs Using Crystal Violet Assay

Further, the effects of AuNPs on biofilm formation with or without nanoparticles were evaluated according to a slightly modified protocol described by O’Toole and Kolter [49]. The detailed method was described in a supplementary file (Material and methods). 

### 4.9. Internalization and Localization of TC-AuNPs: TEM Analysis

The effects of TC-AuNPs on the morphology of bacterial cells and the penetration of TC-AuNPs at sub-MIC into the PAO1 cells were examined by TEM analysis. The PAO1 cells were briefly allowed to grow in the presence and absence of varying sub-MIC of TC-AuNPs. The samples for TEM analysis were prepared by placing a drop of cell suspension in PBS onto the amorphous carbon-coated copper grid and allowing PBS to evaporate at room temperature. After complete drying, the samples were viewed under a transmission electron microscope (JEOL 2100, Tokyo, Japan).

## 5. Conclusions

Biofilm, which is an aggregation of microbial cells, once formed, is difficult to eradicate because it is covered by protective layers of EPS. Therefore, infection caused due to formation of the biofilm is also difficult to treat. In this study, we showed the synthesis of AuNPs using the medicinal plant *T. cordifolia*. The synthesized AuNPs possessed excellent antibiofilm properties against *P. aeruginosa*, as examined by SEM, CLSM, and crystal violet assay. Further, we also concluded that nanoparticles at lower doses are effective against the biofilm of *P. aeruginosa*.

## Supplementary Materials

The following are available online at https://www.mdpi.com/2079-6382/9/3/100/s1, Figure S1: Representation of synthesis of gold nanoparticles using aqueous stem extract of *T. cordifolia*., Table S1: Effects of AuNPs on biofilm forming ability of PAO1., Material and Methods for Characterization of antibiofilm activity of TC-AuNPs using crystal violet assay.
