# Supplementary material for: Biogenic Gold Nanoparticles as Potent Antibacterial and Antibiofilm Nano-Antibiotics against Pseudomonas aeruginosa
Source: Antibiotics (Basel). 2020 Feb 27;9(3):100. doi: 10.3390/antibiotics9030100 (PMC7148532; doi:10.3390/antibiotics9030100)
Supplement: Supplementary file 1 [file antibiotics-09-00100-s001.pdf]

Supplementary data

Material and Methods

Characterization of Antibiofilm Activity of TC-AuNPs Using Crystal Violet Assay

Further, the effects of AuNPs on biofilm formation with or without nanoparticles were evaluated according to a slightly modified protocol described by O'Toole and Kolter [1]. The details method was described in supplementary file. Different tubes (2 mL) were inoculated with the mid-exponential growth phase culture of *P. aeruginosa* (1×10^7 CFU/mL) (100 μL), with or without the nanoparticles, and the tubes were then incubated at 37 °C for 24 h. After incubation, the tubes were washed with distilled water, air dried, and then stained with 0.1% (w/v) crystal violet for 30 min. The crystal violet was then decanted, and the tubes were washed again with distilled water three times, dried, and filled with absolute ethanol. Optical density was recorded at 595 nm.

1. O'Toole, G.A.; Kolter, R. Initiation of biofilm formation in *Pseudomonas fluorescens* WCS365 proceeds via multiple, convergent signalling pathways: a genetic analysis. *Mol Microbiol*. **1998**, *28*, 449–461.

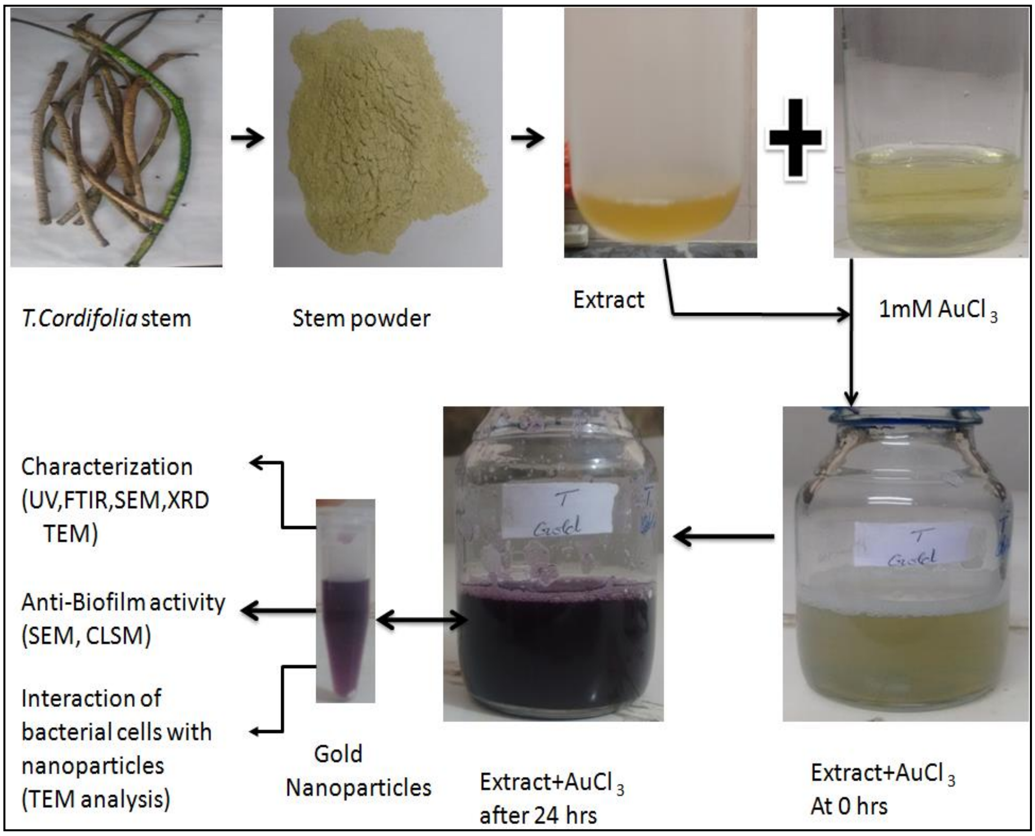

Supplementary Figure S1 Representation of synthesis of gold nanoparticles using aqueous stem extract of *T. cordifolia*.

Supplementary Table S1 Effects of AuNPs on biofilm forming ability of PAO1.

PAO1 (Treated with Gold Nanoparticles)				
	**Treatment(μg/mL)**			
Virulence Factors	Control	50	100	150

Biofilm	2.2 ± 0.12	1.6 ± 0.11*	1.4 ± 0.16*	0.9 ± 0.10*

Biofilm is represented as absorbance at 595 nm; ± means standard deviation; * means significant values $p \leq 0.05$.
